# Draft Genome Sequence of *Paenibacillus* sp. Strain L3-i20, Isolated from Soil in Japan

**DOI:** 10.1128/mra.00077-22

**Published:** 2022-05-16

**Authors:** Daichi Morita, Yoshi Yamano, Sachiko Sugimoto, Katsuyoshi Matsunami, Teruo Kuroda

**Affiliations:** a Department of Microbiology, Graduate School of Biomedical and Health Sciences, Hiroshima University, Hiroshima, Japan; b Department of Pharmacognosy, Graduate School of Biomedical and Health Sciences, Hiroshima University, Hiroshima, Japan; University of Southern California

## Abstract

This study describes the draft genome sequence of *Paenibacillus* sp. strain L3-i20, obtained from an assembly of long reads and subsequently polished using short reads. The draft genome comprises a 5,308,756-bp chromosome with a GC content of 41.6% and no plasmids.

## ANNOUNCEMENT

This microorganism was isolated in the process of attempting to domesticate microorganisms that are difficult to cultivate using a special culture device, as described below, in order to find sources of secondary metabolites that have never been explored before. Genome sequencing was then performed to predict the production potential of the secondary metabolites of the obtained microorganism. The genus *Paenibacillus*, to which the microorganism obtained here belongs, has an abundance of genes for the biosynthesis of secondary metabolites and some isolated compounds, such as polymyxins and fusaricidins, which have been utilized in the field of medicine (1, 2). In this study, we present the draft genome sequence of *Paenibacillus* sp. strain L3-i20, isolated from soil that was collected from the ruins of Nissyoen, Hiroshima, Japan (34°25′N, 132°26′E), in December 2020.

The isolation and cultivation of *Paenibacillus* sp. strain L3-i20 from soil was achieved using a culture device originally created in our laboratory that functions similarly to that developed by Nichols et al. (3) for culturing uncultured microorganisms. For details on the size, materials, and function of the device and the isolation and culture method, see https://doi.org/10.6084/m9.figshare.19336745. To yield genomic DNA, strain L3-i20 was cultured at 25°C for 3 days in Columbia broth, and high-molecular-weight genomic DNA was extracted using a phenol-chloroform extraction technique (4).

The short-read sequencing library was prepared using the NEBNext Ultra II FS DNA library prep kit (New England Biolabs Japan, Tokyo, Japan), according to the manufacturer’s protocols. First, paired-end sequencing (150 bp) was performed using a MiSeq instrument, generating 1,595,849 paired-end reads. Second, the DNA library was prepared for both long- and short-read sequencing using the rapid barcoding kit (Oxford Nanopore Technologies). The prepared library was applied to a FLO-MIN106 R9.41 flow cell (Oxford Nanopore Technologies). Then, the long-read sequences, base called using Guppy high-accuracy mode v.4.2.3, generated 297,005 reads with an average length of 1,541 bp during a 24-h runtime. Next, the low-quality short reads were trimmed using Trimmomatic v.0.39 (5), and the trimmed reads were filtered to remove phiX control sequences using BBDuk v.38.96 (B. Bushnell, BBMap [https://sourceforge.net/projects/bbmap/]). Low-quality Nanopore reads with average Q values of >10.0 were trimmed using NanoFilt v.2.7.1 (6). Using SPAdes v.3.15.2 (7, 8), a hybrid assembly was performed using the short and long reads, and it was polished with the short reads using Pilon (9). Default parameters were used for all software unless otherwise specified.

*Paenibacillus* sp. strain L3-i20 had a genome 5,308,756 bp long, with an *N*_50_ value of 1,244,964 bp and 41.6% GC content. A genome coverage depth of 117-fold was obtained, and the 2 assembled contigs were presumed to comprise the chromosome of this organism. Those *Paenibacillus* species for which whole-genome sequences are available have a single chromosome; therefore, this bacterium is presumed to have a single chromosome. Automatic annotation was performed using the DFAST annotation pipeline (10). Taxonomic estimation and calculation of the average nucleotide identity value of the assembled sequence were performed using HMMER (11) and FastANI (12) by DFAST, respectively, and the closest match was Paenibacillus castaneae (GenBank accession number GCA_002884445.1), with an average nucleotide identity value of 78.7%. Using DFAST, 5,001 coding sequences were predicted.

### Data availability.

The draft genome sequences were deposited at DDBJ/EMBL/GenBank under accession number BQME00000000.2. The raw sequencing data were deposited in the DDBJ SRA database under accession numbers DRR336793 (MiSeq) and DRR336794 (MinION).
